# A Review of the Advancements in the *in-vitro* Modelling of Acute Ischemic Stroke and Its Treatment

**DOI:** 10.3389/fmedt.2022.879074

**Published:** 2022-06-08

**Authors:** Sarah Johnson, Anushree Dwivedi, Mahmood Mirza, Ray McCarthy, Michael Gilvarry

**Affiliations:** Cerenovus (Johnson & Johnson), Galway Neuro Technology Centre, Galway, Ireland

**Keywords:** acute ischemic stroke, mechanical thrombectomy, *in-vitro* modeling, stroke treatment, thrombus

## Abstract

*In-vitro* neurovascular models of large vessel occlusions (LVOs) causing acute ischemic stroke (AIS) are used extensively for pre-clinical testing of new treatment devices. They enable physicians and engineers to examine device performance and the response of the occlusion to further advance design solutions for current unmet clinical needs. These models also enable physicians to train on basic skills, to try out new devices and new procedural approaches, and for the stroke team to practice workflows together in the comfort of a controlled environment in a non-clinical setting. Removal of the occlusive clot in its entirety is the primary goal of the endovascular treatment of LVOs *via* mechanical thrombectomy (MT) and the medical treatment *via* thrombolysis. In MT, recanalization after just one pass is associated with better clinical outcomes than procedures that take multiple passes to achieve the same level of recanalization, commonly known as first pass effect (FPE). To achieve this, physicians and engineers are continually investigating new devices and treatment approaches. To distinguish between treatment devices in the pre-clinical setting, test models must also be optimized and expanded become more nuanced and to represent challenging patient cohorts that could be improved through new technology or better techniques. The aim of this paper is to provide a perspective review of the recent advancements in the *in-vitro* modeling of stroke and to outline how these models need to advance further in future. This review provides an overview of the various *in-vitro* models used for the modeling of AIS and compares the advantages and limitations of each. *In-vitro* models remain an extremely useful tool in the evaluation and design of treatment devices, and great strides have been made to improve replication of physiological conditions. However, further advancement is still required to represent the expanding indications for thrombectomy and thrombolysis, and the generation of new thrombectomy devices, to ensure that smaller treatment effects are captured.

## Introduction

Mechanical thrombectomy (MT) is now firmly the gold standard for the treatment of large vessel occlusions (LVO) causing a stroke and is one of the most effective treatments in medicine (1, 2). However, MT has been rapidly evolving, and the treatment methods and techniques are constantly advancing to achieve better recanalization rates and clinical outcomes.

Preclinical testing using *in-vitro* models has been instrumental in the research and development of thrombectomy devices. Simulations of thrombectomy using transparent and synthetic vascular models with thrombus analogues allows visualization of the interaction between the thrombus, devices, and vessel geometry during thrombectomy (3). This can provide novel insight into therapeutic strategies for efficient removal of clot (4). Although *in-vivo* models remain an ideal environment for such studies, the use of living animals presents many disadvantages, such as high cost, poor reproducibility, and ethical considerations (5). In addition, procedures simulated on animal models often require expensive technical platforms such as dedicated angiographic and general anaesthesia facilities. *In-vitro* models can overcome most of these challenges and provide an opportunity for examining device behaviors in a robust setup. Therefore, these models provide a valuable tool for training, examining device behavior in realistic anatomies and interactions with other devices and clot analogues.

The preferred procedural outcome is achievement of complete recanalization with a single thrombectomy device pass. Recanalization after just one pass is associated with better clinical outcomes than procedures that take multiple passes to achieve the same level of recanalization, commonly known as first pass effect (FPE). To achieve this, physicians and engineers are continually investigating new devices and treatment approaches. For example, larger-bore aspiration catheters for more effective thrombo-aspiration, stent retrievers that are tailored to specific anatomies and/or clot types, or strategies involving various combinations of devices are being developed. Aside from optimizing procedures to achieve FPE in LVOs, treatment of smaller distal vessel occlusions is increasing, which presents additional challenges.

Advancements in AIS treatment technology are required to deliver better patient outcomes, however the treatment effect gained will be smaller compared to the unprecedented clinical improvements seen in the clinical trials that first showed the clear superiority of MT combined with medical management over medical management alone. Clinical trials will need to be larger to capture these smaller treatment effects. Additionally, test models must also become more nuanced and represent challenging patient cohorts to distinguish between treatment devices in the pre-clinical setting. However, many devices currently in clinical use have demonstrated much higher recanalization rates in preclinical models suggesting that current testing platforms and conditions may be oversimplified (6, 7). More realistic modelling platforms may aid the development of devices that would be more likely to achieve first-pass complete recanalization. This perspective review will present the current state of art of *in-vitro* modelling in acute ischemic stroke (AIS) as they relate to large vessel occlusions. Since thrombectomy for LVOs was established only recently as the mainstay treatment, models have been described in non-clinical engineering journals as well as clinical and translational science journals. This article also provides novel insights from current *in-vitro* AIS models and the future technological developments that may lead to more effective and relevant models.

## Characteristics of Current *in-vitro* Models

An ideal *in-vitro* model would simulate realistic device navigation, accurate device-clot-vessel interaction, and replicate the failures seen in clinical practice. To replicate these physiological conditions, the important properties that can affect the model performance are highlighted below.

### Materials

**Figure 1:**
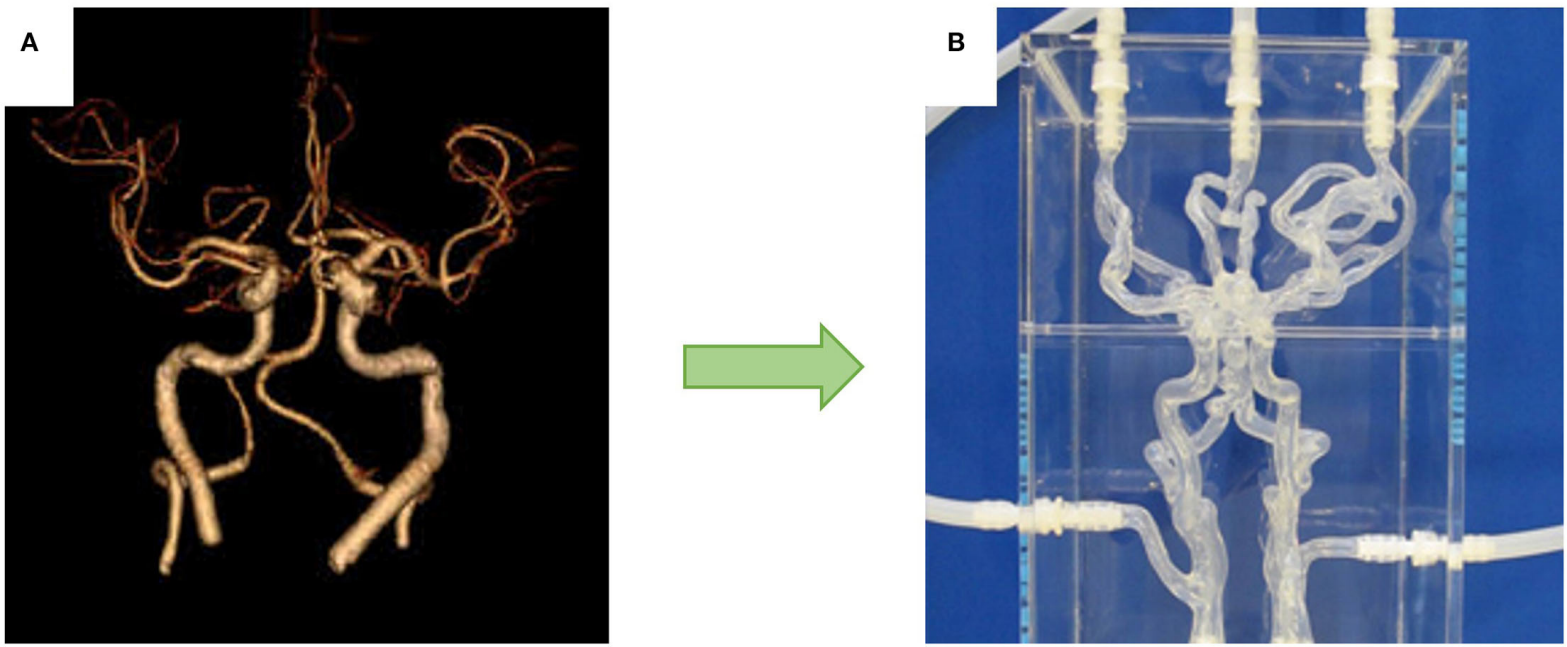
**(A)** Patient Scan which was developed into a **(B)** patient-specific silicone model.

One of the main advantages of many *in-vitro* models is the visualisation allowed by the transparency of the artery replicas, which enables users to observe the clot-device interaction during retrieval. Therefore, complete transparency is the goal for best direct visualization. This can be achieved through glass models, as they have the greatest transparency and clarity, allowing the best visualization of the clot-device interaction (6). They are fabricated by blowing glass tubes for hand shaping. However, due to the fabrication techniques, the glass models may have a more simplified geometry compared with human anatomy. Additionally, glass models are rigid and not compliant like native vessels and therefore do not accurately represent the mechanical behavior and properties. Therefore, silicone is a more commonly used material for *in-vitro* vascular models (8–13). Silicone vessels are transparent and allow direct visual observation of clot-device interaction. In addition, silicone is a robust material that can be moulded to create patient specific and clinically relevant anatomies. Chueh et al. described a small-batch fabrication technique used to produce a population-representative vasculature reconstructed from clinical angiography data (14). In this method, patient- specific vasculature can be reconstructed from CT and then modified to make a core-shell mould with the core having the same geometry as the reconstructed lumen. The mould is 3D-printed and liquid silicone infused into the mould. The whole mould is dissolved after the silicone is cured, resulting in silicone vessels. Other methods involve dipping the core shell in silicone or spraying it on, with subsequent dissolution of the core after curing. Taking advantage of 3D imaging data sets and rapid prototyping techniques, accurate patient-specific vascular replicas (Figure 1) can be fabricated to recreate vessel tortuosity, diameter and length (14, 15). Many of these patient-specific silicone models are now commercially available.

Similarly, direct 3D-printed phantoms have also been fabricated from reconstructed patient-specific anatomies (15). 3D-printed phantoms have shorter lead time to prepare and a lower cost to manufacture. However, a major limitation of these models is their surface roughness and high coefficient of friction. Additionally, transparency of these models is not optimal.

Although these materials provide many advantages for *in-vitro* models in terms of robustness and transparency, the material properties of native vessels are not accurately represented. An important constituent of arterial tissue is its fibrous structure, particularly the arrangement of collagen fibers. These fibers have a major influence on the mechanical behavior and properties of the tissue. The distribution and orientation of the stiff collagen fibers in arterial vessels gives rise to the pronounced anisotropy of the tissue, and therefore the material properties are direction-dependant. This distinguishes the vessel material from typical isotropic materials such as rubber. As well as being anisotropic, the material behavior is also highly non-linear. A typical force-extension curve for arterial tissue demonstrates and initial large extension achieved at low levels of force, followed by rapid stiffening behavior as the collagen fibers become more aligned with the direction of the force (16, 17).

### Friction/Lubricity

The lubricity of synthetic vessels greatly influences device navigation and clot movement. Various methods can be used to alter the coefficient of friction such as surface modification of the vessels or using special fluid to increase lubricity when it interacts with the vessels. However, the characterization of vessel wall friction is lacking, making it difficult to optimize *in-vitro*.

While glass models are rigid and do not represent realistic mechanical behavior of the vessels, their lubricity is high and often better than human vessels (6). On the other hand, 3D-printed models can have very high friction, resulting in more difficult navigation than seen in patients. Vessels made from silicone material can be lubricated to make the coefficient of friction comparable to human vessels. Some investigators have used a liquid silicone rubber coating to mimic the lubricity of the endothelial layer and to reduce the friction on the inner wall of the vessel (14). Kaneko et al. applied ABS solvent to smooth the surface of the model and showed a reduction in the force required to navigate a microwire through the vessel (18). Others have infused the models with slippery fluid and surfactants resulting in similar device navigation compared with patients (15, 19, 20). However, these coatings and lubricants can reduce transparency of these models, causing them to become cloudy in appearance.

Thrombectomy devices apply tensional load (by suction or by pulling the stentretriever) to dislodge the clot, achieved by overcoming the static friction between the clot and vessel wall and the pressure gradient across the clot (21, 22). After the clot is dislodged, the restored blood flow can strip the clot away from the device, leading to re-occlusion or distal embolization. Therefore, *in-vitro* models must accurately replicate the physiological hemodynamics and vessel wall friction in order to accurately replicate these events. While physiological flow has been successfully replicated in the phantoms, the characterization of vessel wall friction is lacking and could be further advanced in future.

**Figure 2:**
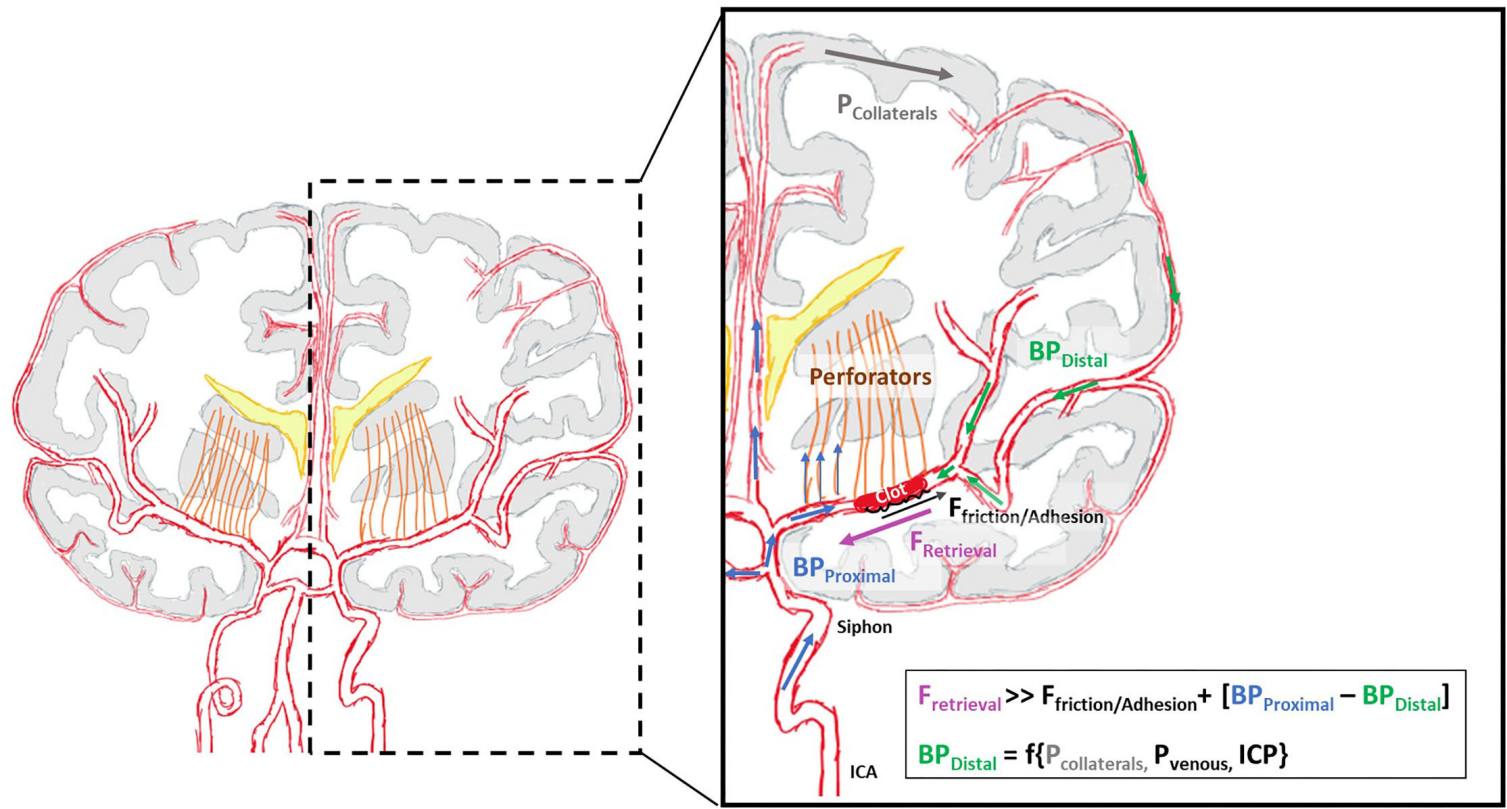
Schematic of forces acting on the clot *in-vivo* during retrieval.

Future 3D printed vascular models may have an additional endothelial lining that could help our understanding of the impact of hemodynamics on the lining of vessel wall. For example, tubular tissue scaffolds where the inner wall is seeded with endothelial cells have been used to compare the endothelial damage caused by aspiration catheters and stentretrievers (SR) (23). Similarly, attempts have been made to grow endothelial cells on the luminal surface by coating the surface with fibronectin (24). Silicone aneurysm tubes have been successfully endothelialized for *in-vitro* evaluation of flow divertors (25). Not only do these advancements allow us to represent the frictional properties of the vessel wall more accurately, but it also allows us to investigate the clot-vessel wall adhesion and interaction (Figure 2).

Recent studies are now exploring the use of 3D printed models to replicate vessel wall pathologies such as stenosis and atherosclerotic plaques (26). Intracranial atherosclerosis disease (ICAD) can complicate mechanical thrombectomy by narrowing the vessel inner diameter (ID). ICAD is also associated with increased procedural risk due the possibility of plaque rupture or embolization during treatment. Adding these pathological features to *in-vitro* phantom models would further improve the accuracy of these models and prevent overestimating the success rate of the mechanical thrombectomy in preclinical tests.

### Compliance

Compliance is the ability of a vessel to dilate and recoil due to the changes in pressure. In accurate vessel movement, expansion, or collapse are primarily influenced by the rigidity and compliance of the material. For example, glass models are rigid, making it difficult to evaluate the response and vessel movement to the mechanical loading of the devices. On the other hand, various grades of silicone have been tested to select a material with a comparable Young's modulus to that of middle cerebral artery (MCA) tissue (14). The anisotropy and fiber orientation can also influence the vessel compliance due to the arrangement of the collagen fibers. Silicone models do allow for some vessel movement during thrombectomy simulation. However, the manufacturing process typically results in a relatively thick wall, reducing the compliance of currently available silicone models.

A greater wall thickness is advantageous for robustness of these models; however, it can be a limiting factor when trying to accurately simulate vessel behavior that is seen clinically. Studies that have utilized fluid–structure interaction (FSI) have reported that rigid wall assumptions can lead to an overestimation of wall shear stress (WSS) in comparison with a compliant phantom (27, 28). In addition, wall stiffness is a measure of arterial health, and the pulse waveform can vary in stiff and compliant arteries. Therefore, failing to mimic arterial compliance can reduce the precision of these models (29).

Although the wall thickness varies in different arterial regions, significant thickness variation in compliant phantoms can cause unrealistic flow fields. Such problems reduce the accuracy of experimental outcomes (28). The arteries of the neuro-vasculature are understood to be thin-walled, compliant vessels. There are many reports in the literature of vessel movement and straightening during thrombectomy (30, 31). There have also been reports of vessel movement and rupture of perforators when treating more distal occlusions due to the higher fragility of these vessels and looser attachment to the parenchyma (32, 33). Thus, a thrombectomy manoeuvre may displace an entire arterial segment, increasing the risk of perforators detaching from the main trunk. As distal occlusions are increasingly targeted for thrombectomy, this may become an important modeling factor to simulate.

Over the past several years, aspiration catheter sizes have been steadily increasing (34, 35) driven by evidence of higher efficacy with larger IDs (8, 20, 36). However, vessels can collapse when the outer diameter of the catheter is close to the inner diameter of the vessel as recently reported during aspiration thrombectomy with a large bore catheter (37). Replicating this phenomenon is important as it would reproduce conditions where loss of flow through an aspiration catheter is caused by vessel collapse rather than clot capture, which may add to procedure time.

Various manufacturing methods have been used for the construction of compliant phantoms (28, 38). However, it is difficult to ensure consistent wall thickness throughout. Additionally, it can be difficult to create thin-walled vessels with small size IDs. For example, the ID of the M1 segment of the MCA can vary from 2 to 3.5 mm in ID. It can be particularly challenging to produce thin-walled, compliant silicone vessels of these sizes. This will become even more difficult as we move towards modelling more distal vessels, which have even smaller IDs. Therefore, significant advancements need to be made in this area to establish methods to reliably manufacture compliant vessels with a small IDs.

### Pressure and Flow

Cerebral autoregulation is well known mechanism to maintain constant overall blood flow to the brain despite changes in cerebral perfusion pressure. However, local blood flow patterns can vary considerably from one person to another. One reason is due to the differences in anatomy, for example due to the level of completeness of the Circle of Willis, level of pial collateralization, or inherent vessel size differences. With modern imaging, the flow rate observed in the MCA ranges from 121 +/- 28mL / min to 144 +/- 43mL/min (39), and the ICA can range from 97.6 +/- 48 (40) to 281 +/- 70.1 mL/min (41). This can greatly impact the behavior of the thrombus under different retrieval thrombectomy techniques, making it a foremost consideration in accurate modeling. Simplified models tend to use continuous flow pumps (42–44), while more recent studies have used programmable pumps that can produce physiologically realistic flow and pressure waveforms in the models (15, 45, 46). Pressure and flow transducers can be used to monitor the flow rates and pressure gradients in the models (20, 44, 45).

When thrombectomy devices interact with the clot, the pressure gradient across the clot can significantly influence the force required to retrieve the clot successfully (Figure 2). During SR thrombectomy, the clot is crossed with the microcatheter and wire, and the SR is deployed across the clot. This crossing of the clot and deployment of the SR may open a flow channel and as a result decrease the pressure gradient for potentially easier clot dislodgement (3, 47). In contrast, in aspiration thrombectomy, the clot is not crossed and thus the pressure gradient remains. The magnitude of the pressure gradient across the clot has been measured in only a few studies (48, 49).

The pressure gradient is thought to be related to patient collaterals (50). Poorer collaterals are believed to be associated with a greater pressure gradient across the clot, due to lower pressure acting on the distal face of the clot, thus contributing to poorer recanalization rates. However further studies need to be carried out in this area to confirm these assumptions. The pressure gradient can also be impacted by the systemic pressure. Further work is required to accurately represent the physiologically relevant pressures and forces acting on the clot during AIS treatment to accurately simulate thrombectomy.

### Circulating Fluid

Viscosity and temperature of the circulating fluid are important considerations to properly simulate aspiration (through aspiration catheters, balloon guiding catheters, with various techniques). It is not generally feasible to use blood as a working fluid in a laboratory setting for many reasons: some of which are health and safety concerns, high opacity, high cost, specific storage requirements, inability to be reused, and its tendency to clot and become unusable. Therefore, blood mimics are used when performing benchtop experiments (51). Saline is a commonly used fluid in *in-vitro* models as it has a similar density to blood (41–43, 50). Glycerol and water mixtures are used to better represent the viscosity of blood (6). The most common ratio is 40:60 glycerol and water, respectively (4, 12, 45). However other ratios such as 50:50 (52), 42:58 (53) and 1:4 (20) have been reported. The viscosity of the blood mimic is of particular importance when simulating aspiration due to the fluid flow through the aspiration catheter during the procedure.

The temperature of the circulating fluid is also a critical factor. It is essential that the circulating fluid is heated to body temperature, approximately 37°C, to ensure the fluid properties are accurately represented at the physiological temperature (54). Additionally, the temperature of fluid can also impact device behavior. For example, SRs made from shape memory metals such as Nitinol are designed specifically to be used at 37°C and therefore their behavior may not be accurately simulated if body temperature is not maintained in the model.

### Anatomy and Vessel Tortuosity

**Figure 3:**
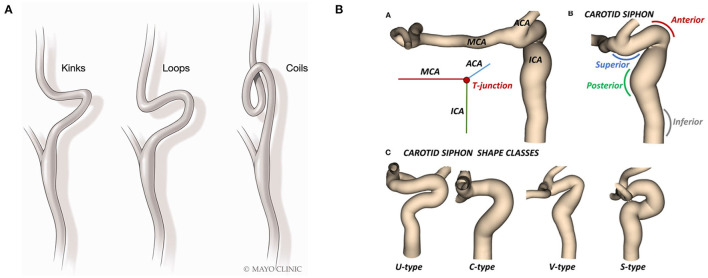
Illustrations of anatomical tortuosity in **(A)** the ICA and **(B)** the carotid siphon. Reproduced from (56, 57) with permission.

Evolution of techniques and devices has greatly increased the success of thrombectomy procedures; however, there remains 15–20% of cases that fail to recanalize sufficiently. Failure to access the occlusion is a significant contributor to failed recanalization. Unfavourable vascular anatomy is a common challenge to neurointerventional procedures. This anatomy may be encountered either in isolation or in combination at many different levels including the aortic arch, common carotid artery (CCA), cervical internal carotid artery (ICA), carotid siphon, and intracranial circulation (30) (Figure 3). In a recent systematic review, it was found that 4.4% of MTs failed to achieve intracranial access using a femoral approach, preventing a single thrombectomy attempt, and leading to revascularization failure. The supra-aortic vessels (2.7%) and aortic arch (1.32%) together account for 92% of the known TFA failure locations, with vessel tortuosity given as the primary reason (2.1%, i.e., 46.8% of all causes) (55).

Challenges associated with ICA tortuosity among patients has long been reported (55, 56). Many categories of tortuosity have been described, including arterial elongation, coils, kinks, and loops. Such anatomical features are common among patients with acute ischemic stroke (AIS), affecting both the intra- and extra-cranial vasculature. Significant variability in ICA geometry can be found in the carotid siphon, which lies between the carotid canal and the ICA-terminus, where the ICA bifurcates into the middle cerebral artery (MCA) and the anterior cerebral artery (ACA] (57). Tortuosity of the ICA has been identified as an impediment to multiple types of endovascular procedures. In patients with AIS, carotid tortuosity may make device delivery more challenging or even impossible, and can cause catheters to kink. Therefore, unfavorable vascular anatomy is associated with worse outcomes and increased revascularization time during thrombectomy (56).

To accurately evaluate device performance *in-vitro*, it is essential to incorporate these anatomical challenges, primarily done through replicating patient-specific imaging scans, though there is room to develop more challenging anatomies. A common tracking challenge for catheters is the distal ICA, which can exhibit sharp bends and branching vessel ostia where catheters can get hung up. Many studies have reported catheters getting caught on the “ophthalmic ledge”—the point at which the ophthalmic artery takes off from the carotid siphon (20, 58). This is commonly referred to as the “ledge effect” (59–61) and can also be seen as catheters get stuck at the posterior communicating artery or the anterior choroidal artery ostia. This challenge is particularly relevant for large-bore aspiration catheters where a larger difference between the inner diameter of the outer catheter and the inner catheter results in a step that catches at bends or branching vessels. The most common technique for mitigating the ledge effect is reduction of the gap between the aspirator and the inner catheter; however, this approach is not effective in some challenging cases (60). Stent-retrievers deployed in a distal vessel can be used as an anchor to help pull the catheter tip away from the vessel obstruction by pulling on the stent retriever wire. Novel, delivery assist catheters have been designed to facilitate delivery of these large-bore catheters to the intracranial circulation, by reducing or eliminating the ledge effect.

Not only are the intra- and supra aortic extra-cranial vasculature important for modeling access challenges, but also tortuous aortic access can delay or prevent access to occlusions. Difficult arch anatomy is the second most common location of failure (55). Challenging arch access is often associated with sharper take-off angle of the innominate or common carotid arteries, or vascular variants such as a bovine arch (62). It is common to encounter tortuosity sequentially at many different levels, i.e., at the arch, in the proximal ICA, and in the ICA siphon. This “stack up” of tortuous anatomies can be extremely challenging when tracking devices to the LVO. Therefore, is it crucial to incorporate tortuous anatomies, from the arch to the intracranial vessels, into *in-vitro* models to predict device performance more accurately.

### Clot

**Figure 4:**
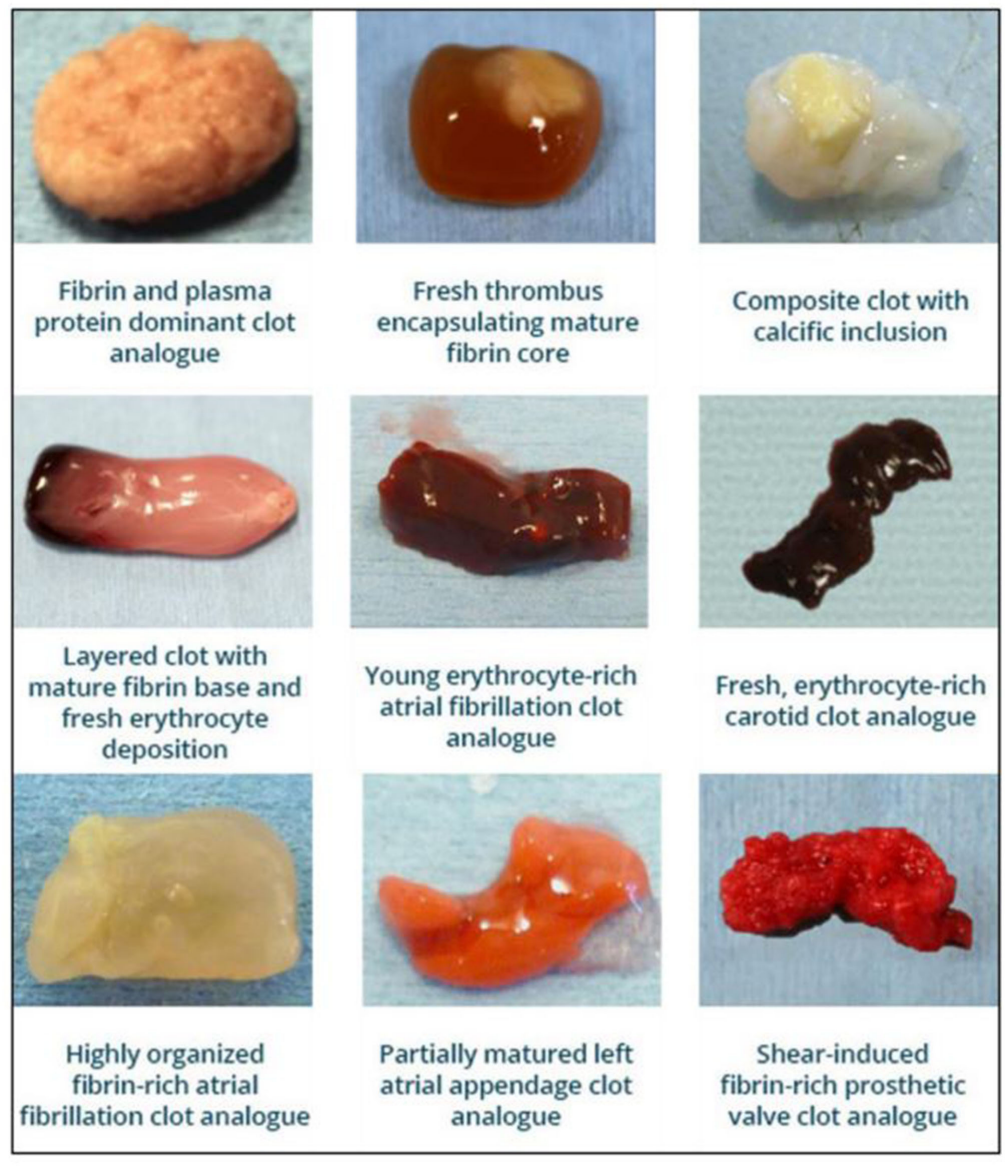
Image of range of clots analogues produced for device testing in *in-vitro* models.

It's now well established that clots causing acute ischemic stroke have a wide variation of composition (63) that leads to an equally wide spectrum of mechanical properties, ranging from soft and easily fragmentable clots to tough and incompressible clots (13, 43, 64–66) (Figure 4). Thus, replicating this variation *in-vitro* is critical to gaining insights into device-clot-vessel behavior. Analysis of thrombus material from human stroke patients has been very instructive, but these thrombi have already been manipulated during the retrieval process. Clot analogues formed *in-vitro* from human or animal blood to simulate the offending blood clots have become increasingly prevalent for the preclinical testing and development of treatment devices as a more appropriate alternative to retrieved human thrombi (67). In comparison to completely synthetic clots from polymers, real blood clot analogues can be produced from real blood, and therefore have a similar chemical structure to native clots. In addition, they are also a useful tool for the investigation and characterization of clot material properties and their mechanical behavior as a function of their constituents.

There are many protocols in the literature to produce clot analogue material. Common variables to generate clot analogues include donor species, the addition of thrombin, the use of radiopacifying media, the variation in haematological components such as red blood cells (RBCs), fibrinogen, platelets, calcium and fibrin content. Modifying any of these components can result in considerably different clot composition and structure (43, 64, 65, 68, 69). For example, the fracture properties of clot material have recently been investigated (70, 71). However, current understanding of the clot morphology reveals differences between clot analogues formed *in-vitro* and the naturally occurring complex human thrombi (64). Research into the morphology of AIS thrombi has revealed that no two thrombi are histologically identical, due to ranging components, such as fibrin and RBC composition (66, 72, 73), presence of neutrophil extracellular traps, von Willebrand factor (74), and platelets (75). Most analogue clot materials have a homogeneous composition, though some heterogeneous clots have been formed (65, 76–78). Chandler et al. proposed forming the clot samples in a constantly moving closed loop to simulate the physiological blood flow environment (77). Clots formed using this method are found to have a fibrin-rich pattern with increased heterogeneity, and are firmer and more elastic in comparison to clots formed under static conditions which are generally RBC dominant when using whole blood (65). Similarly, an experimental approach by Kan et al. prepared the clot analogues by sedimentation to ensure that the clots had a layered structure consisting of a fibrin-rich and an RBC-rich component (78).

It is not always possible to use clot analogues that are derived from blood products due to local regulations. Furthermore, clot analogues derived from blood products are always subject to biological variability associated with normal variations in haematological values as well as a variability associated with techniques to collect blood. Therefore, it may be attractive to have synthetic alternatives.

Robinson et al. prepared moderately stiff synthetic clots using polyacrylamide (67). The clots were used to evaluate the clot capture efficiency of various vena cava filters in a physiological venous flow loop. These devices were also tested using clots formed *in-vitro* from both human and ovine blood. This study found that although the polymeric clots were more convenient and easier to use than the clots formed from blood, there were differences in shape, density and elasticity, and this in turn affected the device performance. Synthetic clot material formed by combining polyacrylamide and alginate (PAAM-Alg) with a crosslinking agent was tested by Merritt et al. (76). Rheometry was used to compare the shear and dynamic moduli of the synthetic polymeric clots with clots formed *in-vitro* using human blood. The PAAM-Alg analogues were found to closely mimic the clots formed using human blood and were easily adjustable to mimic various thrombi classifications.

The growing use of the experimental clot models for thrombectomy device and technique testing continues to inform clinicians with optimum treatment strategies based on laboratory findings, primarily thanks to clinically representative clot analogues (Figure 3). Clot analogues with varying compositions have been used to investigate the effect of clot properties and composition on retrieval success with thrombectomy (8, 20, 43, 44, 79–81). Homogeneous clot analogues can be beneficial in terms of device testing and assessment due to their repeatability; however, they also have limitations as they may not accurately simulate the clot device interactions. An example of this is modeling distal embolization and fragmentation during thrombectomy. Clot debris generated during mechanical thrombectomy or forming *in situ* due to local vessel damage during thrombectomy can result in distal embolization. Heterogeneous clots have been found to be more prone to fragmentation during retrieval and to cause residual occlusion or distal embolization (22). Additionally, *in-vitro* studies have also shown that thrombin-induced clot models can cause thousands of microemboli during the thrombectomy (9, 79).

Several adjunctive endovascular techniques such as balloon guide catheter-assisted SR thrombectomy as well as SR thrombectomy with aspiration *via* a distal access catheter are used in acute stroke interventions to reduce the risk of distal embolization. Therefore, its essential that more realistic and challenging fragmenting clot models are utilized in the testing of thrombectomy devices *in-vitro* to distinguish between techniques.

## Discussion and Recommendations for Future Advancements

*In-vitro* models of the neurovasculature have greatly influenced device development and have been well utilized in pre-clinical testing of new devices and techniques to provide valuable insights into MT effectiveness and failure mechanisms. For example, the NIMBUS device was developed for treating tough clot using data and insights into the clot-device interaction observed from testing performed *in-vitro* (3, 42). Likewise, *in-vitro* models have provided valuable insights into understanding how balloon guide catheter (BGC) positioning and device design can impact distal embolization (79, 80, 82, 83). These models have also provided useful information on the optimal placement of SRs during MT (3) and for the optimization of techniques to remove calcified clots (44).

As AIS treatment technology and devices continue to evolve and develop, treatment indications are also expanding. Therefore, the *in-vitro* models used in the assessment and evaluation of treatment devices should also evolve accordingly. Although mechanical thrombectomy (MT) is now a standard technique in the treatment of AIS in large vessel occlusions (LVOs) with high success rates, there is still considerable scope to improve clinical outcomes. The focus is now largely on the first pass recanalization (FPR)—achieving complete recanalization with a single thrombectomy device pass.

To achieve this, devices and treatment approaches are advancing. For example, larger “superbore” aspiration catheters that are almost occlusive of the vessel ID are being developed for more effective aspiration. Recent studies have shown that the closer the catheter size to the artery lumen (i.e., higher catheter to vessel ratio), the higher the chances of complete recanalization (36, 84). However, larger catheters are more difficult to manoeuvre through tortuous pathways into the intracranial arteries and may cause vasospasms during delivery and vessel collapse during aspiration (20, 85). To distinguish between the performance of these devices on the bench, more advanced *in-vitro* models are required.

Many devices have demonstrated much higher recanalization rates in preclinical models, suggesting that current testing platforms and conditions may be oversimplified (6, 7). This is particularly evident when modelling aspiration thrombectomy. Many *in-vitro* studies report 100% successful recanalization rates when simulating aspiration thrombectomy (8, 20). This is simply not realistic. Therefore, further development in the *in-vitro* modelling of aspiration thrombectomy is required to accurately replicate the challenges and factors that lead to failed recanalization. The next generation of models should represent the physiologically relevant pressures and forces acting on the clot during retrieval so that they are capable of simulating “failed” thrombectomy, particularly with aspiration thrombectomy to more accurately represent the recanalization rates observed clinically.

After MT became widely established, there was some evidence for suggesting direct MT may be favourable to bridging therapy (intravenous thrombolysis followed by MT). This led to clinical equipoise for RCTs, some of which are ongoing, while others are complete. A recent meta-analysis of 6 RCTs suggests direct MT is non-inferior, but perhaps has lower recanalization rates (86). Understanding the influence of thrombolytics on the mechanical properties of clots is limited (87). *In-vitro* models that are capable of incorporating thrombolytics and responsive clots may be helpful in directly investigating their impact to clots and the influence of thrombectomy devices and techniques.

Beyond LVOs, treatment of distal clots as a primary or adjunctive procedure are become more common (88). However, current *in-vitro* models don't include distal vessels and exclusively represent the main cerebral artery lumen and major branching vessels, mainly to the M1 or M2 level. This limitation is primarily due to manufacturing techniques for replicating lumen diameters around 1 mm or less. In addition, vessel movement and straightening of vessels during thrombectomy has been reported (30, 31). As distal arteries are less robust and more loosely attached to the parenchyma, a thrombectomy manoeuvre may displace an entire arterial segment whereby the perforators are at risk for being detached. Devices with a lower radial force or smaller catheters that can traverse highly tortuous fragile vessels may be required to reduce vessel displacement during retrieval.

There are many additional factors, outlined in this paper that can be altered to improve the next generation of *in-vitro* models. Factors such as vessel lubricity and circulating fluid properties will be of particular importance when simulating aspiration as this may affect flow rates through the catheter. Additionally, models with rigid and non-collapsible walls do not necessarily reflect the characteristics of human intracranial vasculature. In real clinical practice, it has been reported that vessels can collapse due to aspiration, especially in the cervical portion (37, 83, 85). This will become even more relevant with the increased use of “superbore” catheters that are more occlusive in the vessel and that have increased suction power when aspirating. It is assumed that vessel collapse between the catheter tip and clot would negate the efficacy of remote aspiration (85), but the degree and extent of collapse of the human intracranial vessels remains poorly studied. Additionally, vessel collapse may also be encountered when treating more distal vessels, which are assumed to be more fragile and compliant. *In-vitro* models with compliant and collapsible vessels would be an extremely valuable tool for the examination of this phenomenon.

The inability to reach or pass an occlusion suggests an insufficient optimization of devices in challenging anatomies. Phantom *in-vitro* models can provide a better option as the geometry can be fully customized. By modularizing the vasculature (from femoral arteries and radial arteries to intracranial arteries) and having different representative anatomies for each module, such models could be used to optimize the navigability of thrombectomy devices with a wide spectrum of anatomical difficulties (6). These tortuous anatomies from the arch to the intracranial arteries will also be essential to include in *in-vitro* models when testing distal occlusion treatment devices and larger bore catheters as the “stack up” of these tracking challenges may impact the device performance.

Alternative options to vascular replicas include cadaveric and animal models. They have been used for testing and training for cardiac and other major vessel procedures but using human cadavers to practice intracranial interventions is challenging. Cadaveric models could theoretically enable optimization of device navigability, but the tortuosity varies for each specimen and a large number is likely required to find challenging anatomies. Another limitation of cadaver models is that circulating blood flow is not present. Therefore, effects such as coagulation and the impact on friction between the devices and vessels may be different from *in vivo* conditions. Furthermore, while the fresh-frozen cadaver model is often considered a “gold standard” for surgical training and biomechanical testing, tissue elasticity and movement may differ from the clinical situation (20).

Likewise, animal models do not recreate the challenges introduced by diseased vasculature found in human. They do not have realistic vascular anatomy or allow detailed observation of device-clot-artery interactions but have the unique value of assessing device safety. However, the main limitation of both models is that repeatability is limited in comparison to *in-vitro* models where many tests can be performed while maintaining consistent test conditions. It is for this reason that *in-vitro* models provide an attractive alternative to animal or cadaveric models as they are more cost effective and can provide a controlled, repeatable environment for device testing and evaluation. With the increasing use of these models as a viable test platform and the recent examples of *in-vitro* models that can represent tissue response (23, 25), this may significantly reduce and possibly eliminate the need for animal studies for device testing in future.

Recent studies have suggested using human placenta as an *ex-vivo* vascular model for neurointerventional surgery research and training purposes due to its vessels resembling the brain vasculature. Similarities were found in vascular branch patterns, histological cross sections, and angiographic appearances (89). Due to the semitransparency of its vessel wall, devices can be visualized under direct microscopic observation. Endothelial change after thrombectomy in these placenta models may also be assessed by histological methods. The model opens doors for *in-vitro*/*ex-vivo* testing of new endovascular devices during the development phase in the future. Ideally *in-vitro* and *ex-vivo* models should be used in combination to complement each other to acquire a comprehensive understanding of the failure modes of MT devices.

In addition to physical models, computational modeling is gaining attention and likely to have an increasing role in medical device development (57, 90–92). Finite element modeling is recommended by the FDA, in the guidance for non-clinical testing of stents and associated delivery systems, as a means of performing stress and fatigue analysis on the devices seeking approval (93). However, this recommendation is currently not included in the guidelines for non-clinical testing of neurothrombectomy devices. As these devices are emerging as the new standard of care for stroke treatment, it is possible that the criteria for FDA approval may become more rigorous in the future, and this may lead to the requirement of finite element analysis of these devices. Finite element analysis is also a powerful tool used in the medical device industry to analyze performance of devices during development. However, in order to perform finite element analysis of these thrombectomy devices, an accurate constitutive representation of thrombus material (69).

Recent publications from the INSIST project (IN- Silico trials for treatment of acute Ischemic Stroke) report the development of a platform where computational simulations of thrombosis and thrombolysis, thrombectomy, perfusion, and brain tissue infarction are carried out on “virtual patients” (57, 94). These “virtual patients” include clinically representative characteristics such as clot properties, vessel geometries, and clinical record. However, accurately modeling the complex physiology, anatomy, and arterial response to mechanical loads remain a daunting challenge and validation of these models is crucial before one can make use of their great potential for analyzing and improving clinical treatment procedures and devices.

**Figure 5:**
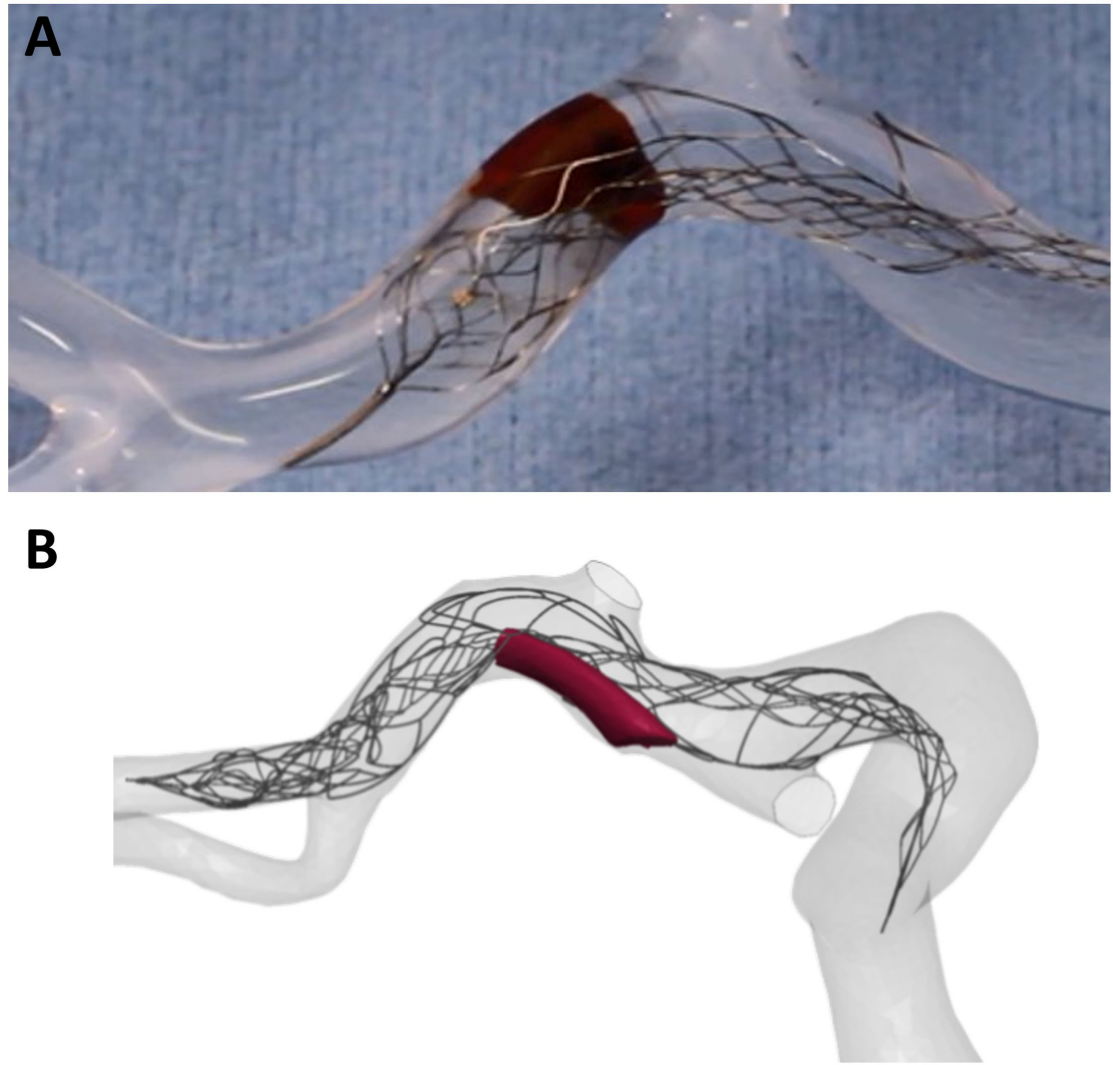
Image of **(A)** *in-vitro* modelling of thrombectomy and **(B)** finite element *in-silico* simulation of thrombectomy. *In-vitro* models provide a great platform for this crucial validation step of *in-silico* models.

One major challenge for *in-silico* models is the development of policy and procedures around proving credibility of these models and *in-silico* trials *via* validation, verification, and uncertainty quantification (VVUQ) practices (95). Currently, the models are undergoing independent validation activities, using both clinical and in vitro testing (90). For example, device test data and previously characterized clot material properties were utilized by Luraghi et al. in the development of finite element models of stent-retriever thrombectomy (90). *In-vitro* thrombectomy tests were also performed and were used for the validation of the *in-silico* simulations (Figure 5).

In conclusion, experimental models of ischemic stroke are valuable tools to analyze specific facets of stroke and stroke treatment. However, being aware of the limitations of the individual models and ideally having support from additional models and studies using are of utmost importance before drawing conclusions concerning stroke in humans (96). *In-vitro* models also provide a crucial validation step of *in-silico* models and the combination of both *in-vitro* and *in-silico* models are essential tools for the assessment of the existing devices and procedures and also for designing new devices and optimization of treatment. Likewise, combining *in-vitro* and *in-vivo* models also provide a greater understanding of AIS and this combination may even significantly reduce and possibly eliminate the need for animal studies for device testing in future.

## Conclusion

The primary aim of *in-vitro* stroke modeling is to verify and validate emerging new technologies and treatment options in a way that can translate accurately into the clinical setting. *In-vitro* models remain an extremely useful tool in the evaluation and design of treatment device, and great strides have been made to improve replication of physiological properties, challenging anatomies, and the offending clot. However, further advancement is required to represent the expanding indications for thrombectomy and thrombolysis, and the generation of new thrombectomy devices, including more realistic anatomy, friction, flow conditions, and vessel wall response to ensure that smaller treatment effects are captured.

## Author Contributions

SJ: brainstorming, preparation, writing, and review of manuscript. AD: writing and review of manuscript. RM and MM: brainstorming, writing, and review of manuscript. MG: brainstorming and review of manuscript. All authors contributed to the article and approved the submitted version.

## Conflict of Interest

The authors were employeed by Cerenovus (Johnson & Johnson).

## Publisher's Note

All claims expressed in this article are solely those of the authors and do not necessarily represent those of their affiliated organizations, or those of the publisher, the editors and the reviewers. Any product that may be evaluated in this article, or claim that may be made by its manufacturer, is not guaranteed or endorsed by the publisher.
